# Morphometric characteristics and seasonal proximity to water of the Cypriot blunt-nosed viper *Macrovipera lebetina lebetina* (Linnaeus, 1758)

**DOI:** 10.1186/s40409-018-0175-6

**Published:** 2018-12-27

**Authors:** Daniel Jestrzemski, Irina Kuzyakova

**Affiliations:** 1 Faculty of Forest Sciences and Forest Ecology, Department of Forest Zoology and Forest Conservation, University of Göttingen, Büsgenweg 3, 37077 Göttingen, Germany; 2 Faculty of Forest Sciences and Forest Ecology, Department of Ecoinformatics, Biometrics and Forest Growth, University of Göttingen, Büsgenweg 4, 37077 Göttingen, Germany

**Keywords:** Cyprus, Blunt-nosed viper, Morphology, Body condition index, Ecology, Water, Snakebite, Conservation

## Abstract

**Background:**

The blunt-nosed viper *Macrovipera lebetina* (Linnaeus, 1758) is a medically important snake species in the Middle East. Its nominate subspecies *Macrovipera l. lebetina* is confined to Cyprus, where it is the only dangerously venomous snake species and heavily pursued. Despite the viper’s large size, data on its body mass and sex-specific morphological differences are scarce. It is commonly believed that *M. l. lebetina* prefers freshwater proximity during summer. Hence, we aimed at investigating *M. l. lebetina* sex-specific morphological differences and its possible attraction to freshwater bodies in late summer.

**Methods:**

Morphometric characteristics, proximity to water and conservation status of *M. l. lebetina* were investigated in Paphos district (Cyprus) in 2014, 2015 and 2017. Vipers were caught in different habitats, examined morphologically for metric and meristic characters, and released back into their habitat. Additionally, local people were interviewed about the conservation situation of the species.

**Results:**

Of 38 recorded blunt-nosed vipers, morphological characteristics were collected from 34 (10 adult males, 16 adult females, eight unsexed juveniles). Rounded total length (ToL) ranged from 23.5 cm to 133.0 cm and weight between 10 g and 1456 g. Adult males significantly exceeded adult females in tail length (TaL), ToL and head length (HL). No significant sex-specific differences were found in snout-vent length (SVL), head width (HW), weight or body condition index (BCI), nor for the ratios TaL / SVL, TaL / ToL, HL / SVL or HL / HW. Adult females from late summer (2015) had a significantly lower mean BCI than those from spring (2014).

Distances of blunt-nosed vipers to the nearest water bodies (natural and artificial, respectively) did not differ significantly between spring (2014) and late summer (2015). There was also no significant difference between the distances of vipers to natural and to artificial water bodies in spring (and late summer).

**Conclusions:**

Adult male blunt-nosed vipers exceed adult females in TaL, ToL and HL. Adult females are likely in a more vulnerable body condition in late summer than in spring. Periodic drying out of freshwater bodies in summer probably does not affect the species’ occurrence. Educational workshops and habitat conservation are recommended for reducing human-viper conflict.

## Background

In the Near and Middle East, true vipers (Viperinae) are of significant medical importance [1–5]. The blunt-nosed viper *Macrovipera lebetina* (Linnaeus, 1758) for instance has been a cause of serious snakebite accidents from Cyprus and Eastern Anatolia to Northwest India [1, 6–11], while its venom is of high value for drug research and development [12–16]. *Macrovipera lebetina* inhabits river valleys, slopes with rocky outcrops and shrubs as well as canyons, gorges, gullies, pine forests, orchards, vineyards, caves and ruins [8, 17]. This large, heavy-bodied viper is an ambush predator of small mammals and birds and is often found in agricultural areas where it feeds on rodents [8]. The nominate subspecies *Macrovipera lebetina lebetina* (Cypriot blunt-nosed viper) is confined to Cyprus [8]. It is the only viper species found on the island and the largest viper in the European Union, growing up to 150 cm in total length (ToL) [17]. Blunt-nosed vipers from the mainland (e.g., *M. l. obtusa*) may even exceed 200 cm in ToL, with the published record being 230 cm [18]. However, the average ToL is between 80 and 100 cm for most *M. lebetina* populations [19]. According to Ščerbak & Böhme (2005), the largest blunt-nosed viper subspecies (*M. l. turanica* in Central Asia) reaches a ToL of 180 cm. The maximum weight of *M. lebetina* is 2700 g [8]. Despite the special status of *M. l. lebetina* within the European viper fauna, little information is available on the natural history of this subspecies. Furthermore, no body mass data have been published yet on the Cypriot blunt-nosed viper. Although snake body mass (or weight) is rarely reported in the literature, it is considered the best measure of body size in life history studies and often a more useful proxy than length [20]. In this study, the snake body condition index was calculated as a measure of thickness based on *M. l. lebetina* body weight and length [21, 22]. As published data on sex-specific morphological differences in *M. lebetina* are scarce and sometimes contradictory, comparing different morphological characteristics between male and female blunt-nosed vipers can contribute to filling this knowledge gap. Based on the literature [8, 17], we formulated and tested three hypotheses: (1) On average, adult males are larger in snout-vent length (SVL) than adult females. (2) The ratio of tail length to snout-vent length (TaL / SVL) is smaller in adult males than in adult females. (3) In late summer (after the mating season), adult females are thinner than in spring (during the mating season).

Reptiles in semi-arid regions are often confronted with annual droughts. The resulting periodic water scarcity affects their spatial distribution, foraging ecology and body condition. Water availability is an important factor for survival as it may also determine the presence or absence of prey species and shelter from overheating and natural enemies [23, 24]. As water bodies are critical for sustaining both wildlife and human civilization, they have been facilitating contact between people and reptiles for millennia. In some areas, this contact has contributed to the problematic relationship between humans and venomous snakes. During the summer months, when many natural water bodies dry out in Cyprus, *M. l. lebetina* is frequently observed close to water [17, 25] and can even be found in swimming pools. Bites have occurred at waterholes [17]. Although a better understanding of *M. l. lebetina* distribution patterns and habitat requirements can contribute to minimizing human-viper conflicts, little is known about the importance of water for the spatial ecology of this species. In this study we therefore also investigated the relationship between water occurrence and blunt-nosed viper presence in Cyprus, via analysis of distances of the vipers to the nearest body of artificial and (or) natural freshwater body.

## Methods

### Data collection

**Fig. 1:**
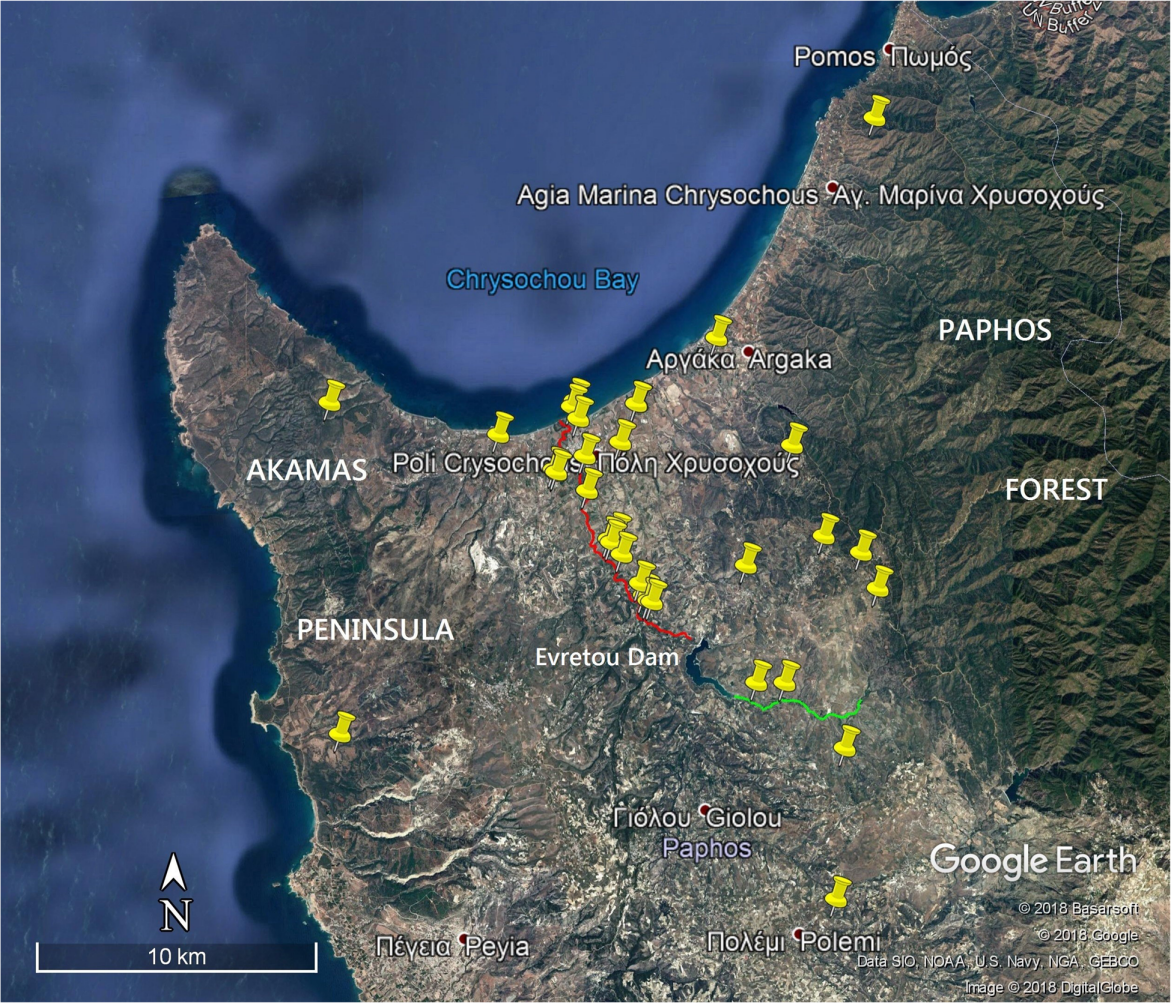
The study area in northern Paphos district: Polis Chrysochous municipality (35°02’ N, 32°26’ E) and surroundings, bordered by Akamas Peninsula in the west and Paphos Forest (Troodos Mountains) in the east. The Chrysochous River is highlighted in red. The lower section of the Stavros tis Psokas River, which flows into the Evretou Reservoir, is highlighted in green. The yellow pin markers represent the locations of the recorded *M. l. lebetina* specimens from this study, with one individual from near Kakopetria (Nicosia district) not included

Data collection took place during three field surveys in northern Paphos district, Cyprus (ca. 35°02’ N, 32°26′ E), from 24 March to 4 June 2014, 27 August to 29 September 2015 and 21 September to 3 October 2017. This coastal lowland area is characterized by agriculture in the Polis Chrysochous valley, bordering Akamas Peninsula in the west and the forested foothills of the Troodos Mountains in the east (Fig. 1, see [26, 27]). Daily mean temperatures range from 12.1 °C in January to 27.6 °C in July and August, with maximum temperatures of ≥40 °C during summer. Mean annual precipitation is 394.2 mm, of which 380.9 mm falls between October and April, and 13.3 mm between May and September [28]. Potential viper habitats in Polis municipality and surroundings were systematically examined via a transect survey [29]. Sixteen transects were placed across different potential viper habitats classified as (1) edges along streambanks and water pools, (2) edges along grain fields, orchards and pastures, (3) agrarian landscape mosaic with ruins of a deserted village, (4) shrubby slopes (maquis and garrigue habitat) in eastern Akamas Peninsula. Over each habitat, 1500 m of transect line was spread, divided into three 500-m-long sections. Additionally, field trips were made to nearby gorges (Androlikou, Petratis and Avakas) and dammed reservoirs (Evretou, Argaka, Agia Marina and Kannaviou), as well as to western Akamas Peninsula and the Troodos Mountains. Road-cruising was applied as a further survey method. Diurnal transect surveys amounted to 8 hours h daily throughout spring (starting at 8:00 AM), and nocturnal surveys to 4 h daily in late summer (starting at 20:00 PM). During late summer, daily diurnal survey time was reduced to 4 h in the morning and early evening time. Three to four days were needed to examine all 16 transects (including field data collection), before repeating the process. Transect lines were walked in both directions and carefully checked for blunt-nosed vipers within a range of 20 m. At every viper observation point, the date, time, climatic and GPS data were collected and the shortest distance to the nearest water body was measured. Distances were measured with a 30 m tape in the field or otherwise on the computer with the Google Maps distance calculator. Swimming pools and other man-made reservoirs supplied by piped water were defined as artificial water bodies, whereas streams, natural springs, reservoirs and lakes were considered natural water bodies. Live individuals of *M. l. lebetina* were caught for the collection of biometric data. Body weight was measured to the nearest gram (mean of five measurements) using a Beurer digital scale. Snout-vent length, tail length (TaL) and ToL were measured to the nearest 0.5 cm in live individuals and to the nearest 1 mm in dead ones. For statistical purposes, SVL, TaL and ToL of dead specimens were later rounded to the nearest 0.5 cm. Head length (HL) and head width (HW) were measured to the nearest 1 mm. Ventral, subcaudal and dorsal scales were counted, the latter at mid-body. The sex was determined via examination of the tail base and cloaca in live specimens, and via probing of the hemipenes in dead individuals [17]. Vipers below 70 cm ToL were considered sub-adult [11]. To avoid ambiguity in sex determination and risk of injury, juveniles were not sexed. Following Benson (1999), a digital photograph of the anal plate of each specimen was taken for individual recognition [30]. After being kept in captivity for up to 24 h, live individuals were relocated to their native areas, or, if captured in residential areas, to the nearest appropriate habitat. Four specimens (SNM-BS N-56085 to N-56088) found dead in good condition were collected and stored in 90% ethanol. They were later transferred to Braunschweig State Natural History Museum (Germany) and dissected for a stomach content analysis. In addition to these morphological and ecological data, semi-structured interviews on human interaction with *M. l. lebetina* were conducted with staff of Cypriot institutions and local people related to environmental management, outdoor labor and medicine.

### Statistical analysis

For all collected morphometric measurements, the mean, standard deviation (SD) and coefficient of variation (CV) were calculated. As a body condition index (BCI) for each snake, Fulton’s index (K) was calculated using the formula $K=\frac{Weight}{SV{L}^3}\mathrm{x}\ {10}^5$ [21, 22]. Prior to statistical analysis, all distance and morphometric data were tested for normality using the Shapiro-Wilk test. Due to non-normality of the distribution, the Mann-Whitney U test was applied to compare the nearest distances to water in the spring of 2014 with those taken in late summer of 2015. For the comparison of the nearest distances to artificial and natural water bodies in the spring of 2014 (late summer of 2015), the Wilcoxon matched pairs test was applied. Relationships between morphometric characteristics were examined via regression and correlation analysis. We assumed that the relationship between SVL and weight (W) could be described by the exponential regression equation *W* = *aSVL*^*b*^, where *a* and *b* are constant [31]. The coefficients *a* and *b* were estimated for the linearized form of the equation (after log-transformation of both sides of the equation) *logW* = *loga* + *bSVL*, using the least squares method. The coefficient of determination (R^2^) was used as a measure of the fraction of weight variation that can be explained by SVL. The correlation coefficient R was used as a measure of the strength of the linear relationship between SVL and TaL, SVL and HL, SVL and HW, and between HL and HW. Morphometric properties and the BCI of adult males and females were compared using the unpaired *t* test for normally distributed variables or the Mann-Whitney U test for non-normally distributed variables. For all tests, results were considered to be significant when *P* < 0.05. All statistical analyses were performed using the software Statistica 13.2 (StatSoft, Inc., USA).

## Results

### Habitat

*Macrovipera l. lebetina* was observed at edges of agricultural plantations, grain fields, gardens and streambanks, on grassy slopes, and inside dried-out streambeds. Besides shrubs and thick grassy vegetation, habitats with confirmed blunt-nosed viper presence contained a variety of microstructures such as crevices and caves, animal dens, stone slabs, rock piles, rock walls, and ruins of buildings and other constructions. Ten blunt-nosed vipers were recorded in private gardens (April 2014, July and September 2015), two in a building next to a swimming pool (3 and 19 May 2014) and a killed (newborn) viper at a building entrance door (21 September 2015). An adult female was found inside a pickup truck into which it had crawled when the vehicle had briefly stopped on a dirt road (23 September 2015). Common plant species of surveyed *M. l. lebetina* habitats in Paphos district included oriental alder (*Alnus orientalis*), giant reed (*Arundo donax*), common fig tree (*Ficus carica*), Phoenician juniper (*Juniperus phoenicea*), olive tree (*Olea europaea*), lentisk (*Pistacia lentiscus*), oriental plane (*Platanus orientalis*), holy bramble (*Rubus ulmifolius sanctus*) and the exotic eucalypt (*Eucalyptus* spp.).

### Food habits

**Fig. 2:**
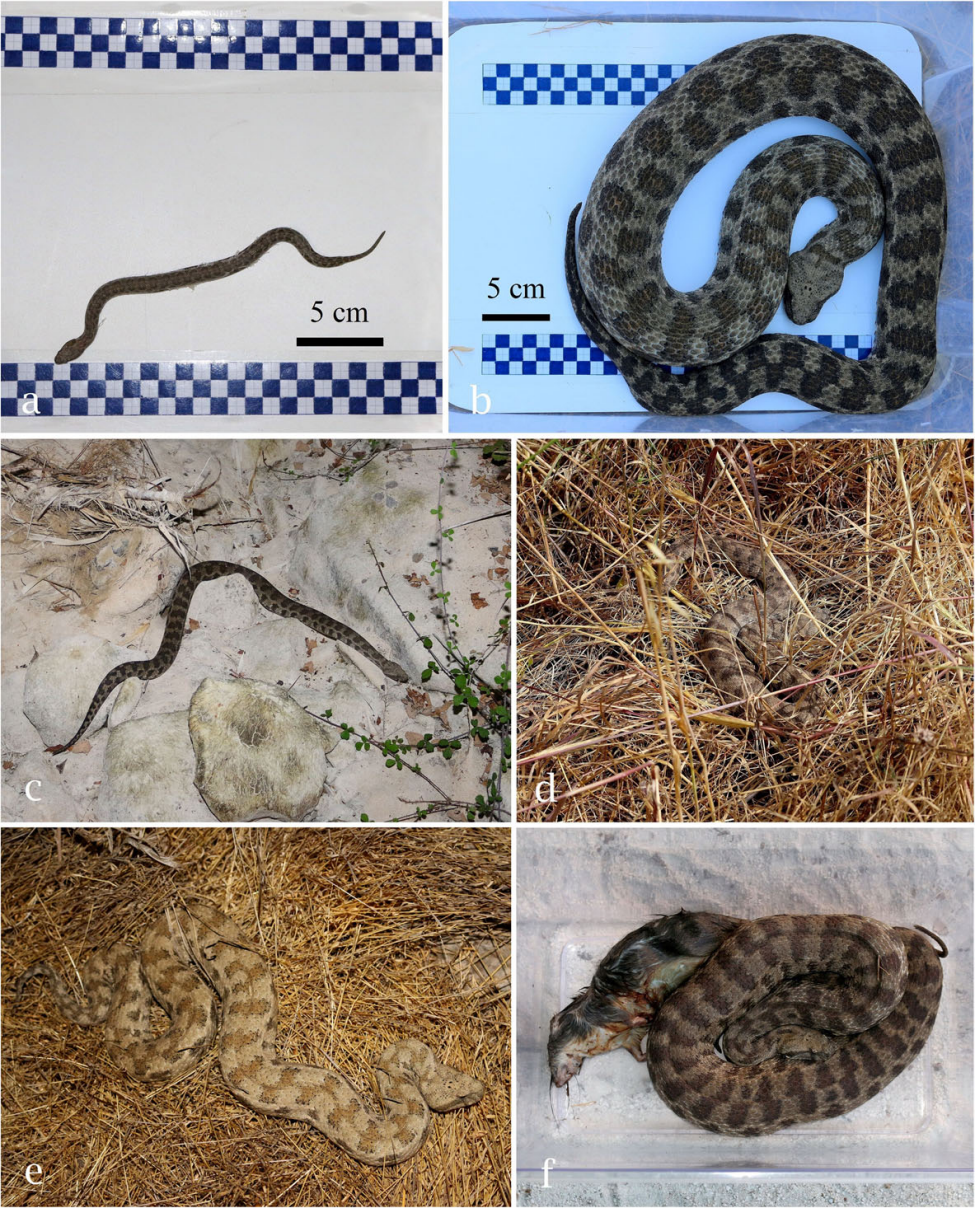
**a** Dorsal view of a neonate blunt-nosed viper (SVL: 20.5 cm, TaL: 3 cm, weight: 10 g) with a 5 cm scale (11 September 2015). The skin is partially shed. **b** Dorsal view of an adult male blunt-nosed viper (SVL: 116.5 cm, TaL: 14.5 cm, weight: 1441 g) with a 5 cm scale. **c** The individual from 2b in a periodically dry streambed where it was found in ambush position on 6 September 2015, 22:05 PM. **d** Young adult female blunt-nosed viper (SVL: 65.5 cm, TaL: 9 cm, weight: 279 g) basking well-camouflaged at the edge of cropland after rainfall (6 May 2014, 15:45 PM). **e** Adult male blunt-nosed viper from Akamas Peninsula (SVL: 92.5 cm, TaL: 12.5 cm, weight: 749 g), found in maquis and garrigue shrubland (28 September 2017, 20:45 PM). **f** Adult female blunt-nosed viper (SVL: 86.5 cm, TaL: 11.5 cm, weight: 835 g) with a regurgitated brown rat (*Rattus norvegicus*) of 270 g. Found moving along a stream bank (Fig. 3a). Photos: D. Jestrzemski

The stomach of an adult female blunt-nosed viper (SVL 109.0 cm, TaL 14.8 cm, weight 1225 g; SNM-BS N-56086) contained remains of a tawny pipit (*Anthus campestris*). This snake was found killed on a country road (10 April 2014). A live adult female (SVL 86.5 cm, TaL 11.5 cm) caught on 1 May 2014 (11:07 AM) at the edge of a stream bank next to an olive tree plantation regurgitated a half-digested, adult brown rat (*Rattus norvegicus*) of 270 g after 5 h in captivity (Fig. 2f). After regurgitation, the body weight of the snake had dropped from 1123 to 835 g. The skull of a broad-toothed field mouse (*Apodemus mystacinus*) was found in the stomach of a killed adult male viper (SVL 92.3 cm, TaL 13.2 cm, weight 730 g; SNM-BS N-56087) lying on a small village road (12 May 2014). A killed male found on 17 September 2015 (SVL 117.3 cm, TaL 14.5 cm, weight 1302 g) contained remains from a rodent, most likely a brown rat.

### Shedding locations

Two blunt-nosed viper shedding localities were recorded during the field survey. On 1 April 2014, the slough of a large adult viper (about 1.2 m ToL) was found at the edge of an orange tree plantation and grazing pastures interspersed with almond trees. The slough was in the grass next to a pile of wooden debris. Two adult females (SVL = 65.5 and 93.0 cm) were later observed 33 m (6 May 2014) and 119 m (2 September 2015) away, respectively. The distance of the slough to the next water body (an artificial, permanent reservoir) was 137 m. The slough remnant of another large adult blunt-nosed viper (including a 15.5 cm long tail section) was discovered inside a periodically dry streambed with several small water pools on 27 September 2015. In the same month (6 and 29 September), two large vipers were recorded 76 m and 67 m upstream from this shedding location. The individual of 6 September was an adult male (SVL = 116.5 cm, TaL = 14.5 cm).

### Bite accidents by *Macrovipera lebetina* in Cyprus

**Fig. 3:**
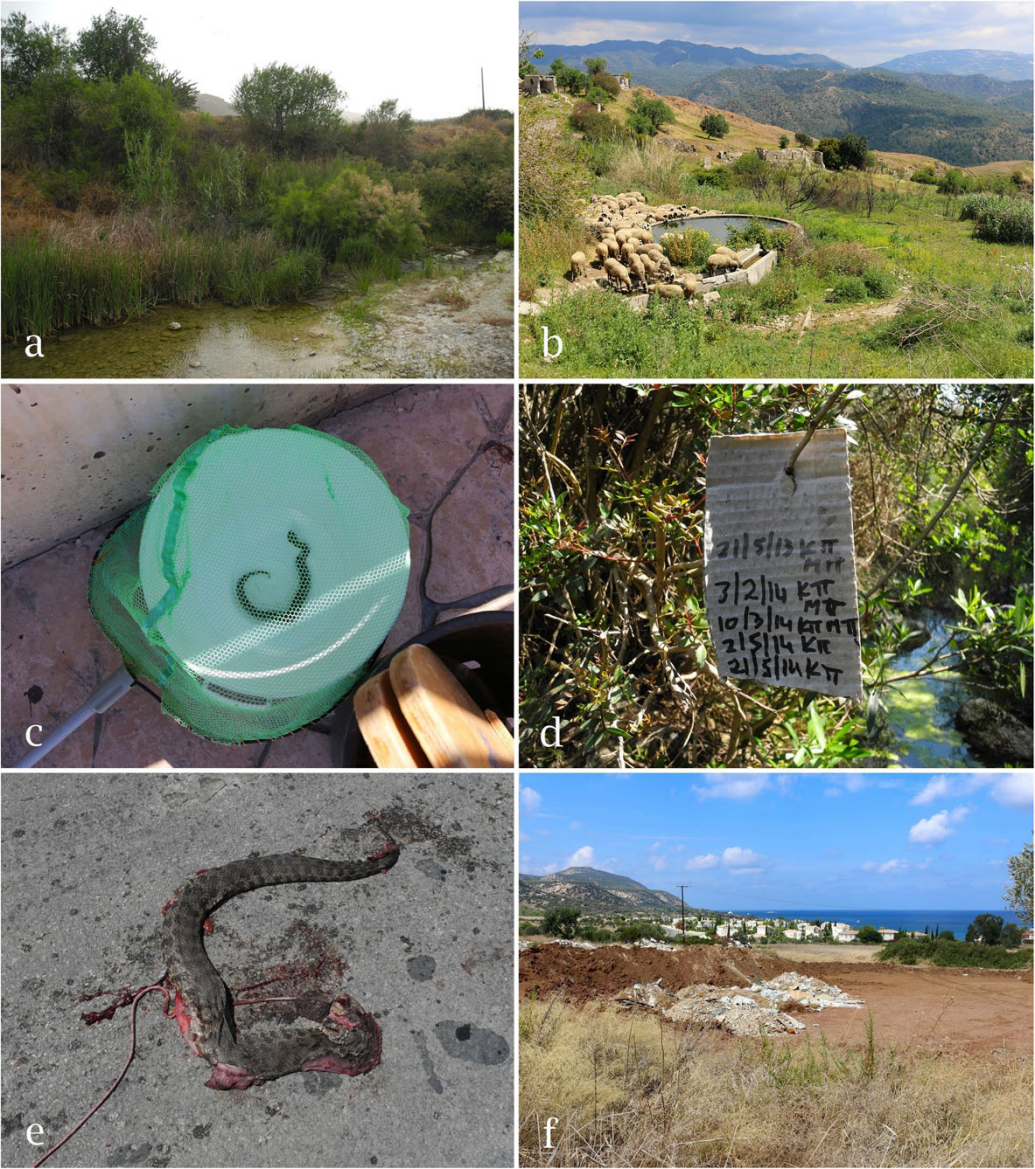
**a** Richly structured stream bank with adjacent agricultural areas: habitat of the blunt-nosed viper. **b** Rocky slopes with shrubs and ruins: blunt-nosed viper habitat used for sheep and goat grazing. **c** Juvenile blunt-nosed viper caught by children at noon (12:40 PM) in a residential swimming pool in central Paphos district (13 September 2015). **e** Signatures of mosquito control workers at a stream show that this riparian area – habitat of *M. l. lebetina* – is regularly visited by people. **e** Blunt-nosed viper killed and dragged on the road with a string attached to the snake’s neck (14 April 2014). **f** Destruction of wild blunt-nosed viper habitat for real-estate development on Akamas Peninsula (1 October 2017). Loss of pristine habitats is a common problem in coastal areas of Cyprus. Photos: D. Jestrzemski

During the field study, news was received of at least four incidences of human envenoming following snakebite in the study area. In one case (on 15 September 2015), a woman was bitten on her foot by a juvenile blunt-nosed viper next to her swimming pool (S. Paphitis pers. comm. 17 September 2015). As a survey of snakebite epidemiology was beyond the scope of the present study, it is possible that additional people in the area might have been bitten by vipers during the study period. Livestock and hunting dogs are bitten by *M. l. lebetina* every year (H.-J. Wiedl pers. comm. 29 March 2014; K. Kailis pers. comm. 15 April 2014; H. Demetriades pers. comm. 29 May 2014). In summer, bite accidents happen especially when goats and sheep concentrate around waterholes frequented by vipers (H. Hadjistyllis pers. comm. 6 June 2014; Fig. 3b).

### Conservation status

During the 2014 survey (between 3 April and 26 May), 13 blunt-nosed vipers were found killed on roads in Paphos district (Polis area) with injuries including smashed heads, necks and cloacae. In two cases, a string was attached to the body of the snake (Fig. 3e). On 17 September 2015, a large dead male was found underneath the edge of a road bridge in a dried-out streambed, with its skull smashed and its stomach content protruding from its open gut. Interviews confirmed that *M. l. lebetina* is feared and persecuted by local people both in populated areas and in the wild. Road traffic is another threat. People involved in outdoor activities (farmers, forestry employees, game wardens, hunters, mosquito control workers and shepherds) encounter vipers more often in comparison with other occupational groups. The widespread public aversion to *M. l. lebetina* has so far prevented legal protection of this species in Cyprus ([17]; E. Erotokritou, H. Nicolaou pers. comm. 28 May 2014). Additionally, wild viper habitat is continuously destroyed by real-estate development, an ongoing trend in coastal areas (pers. obs., Figure 3f). Man-made wildfires occur every year. In June 2016, 751 hectares of vegetation were burned down in the Argaka area (H. Nicolaou pers. comm. 17 March 2018).

### Morphometric measurements

**Fig. 4:**
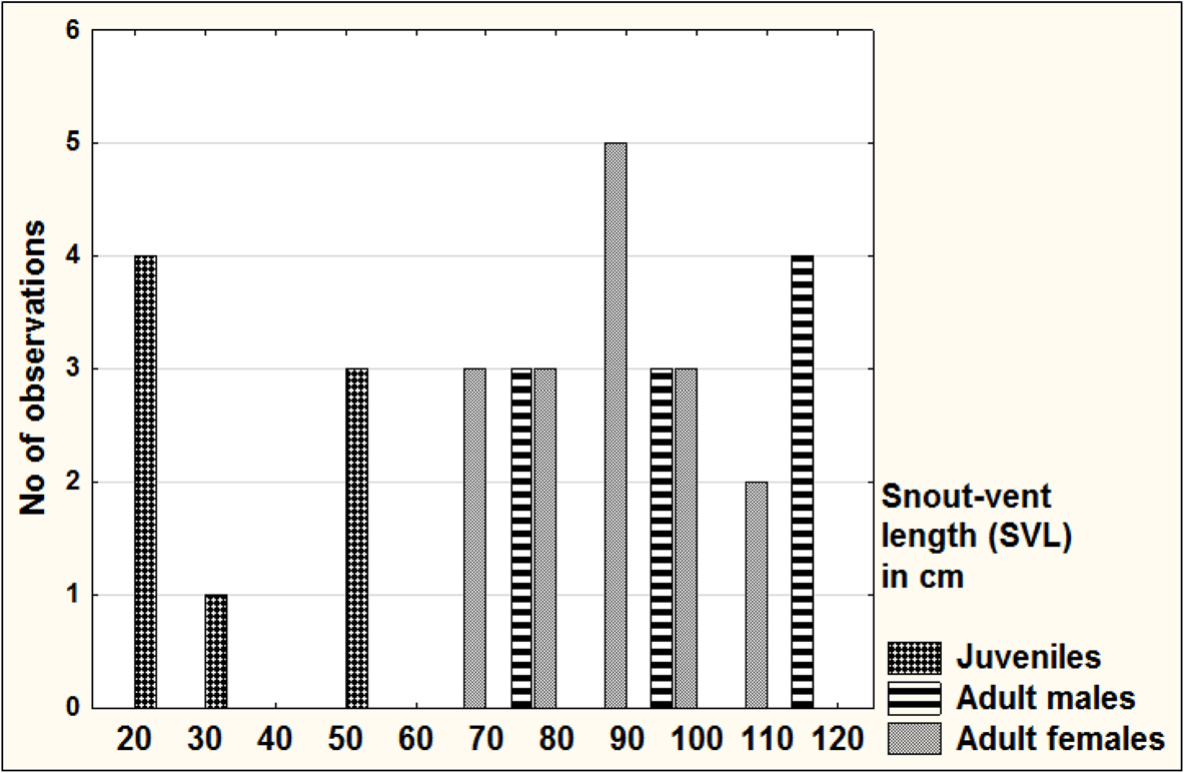
Distribution of SVL among 34 blunt-nosed vipers from Cyprus

**Fig. 5:**
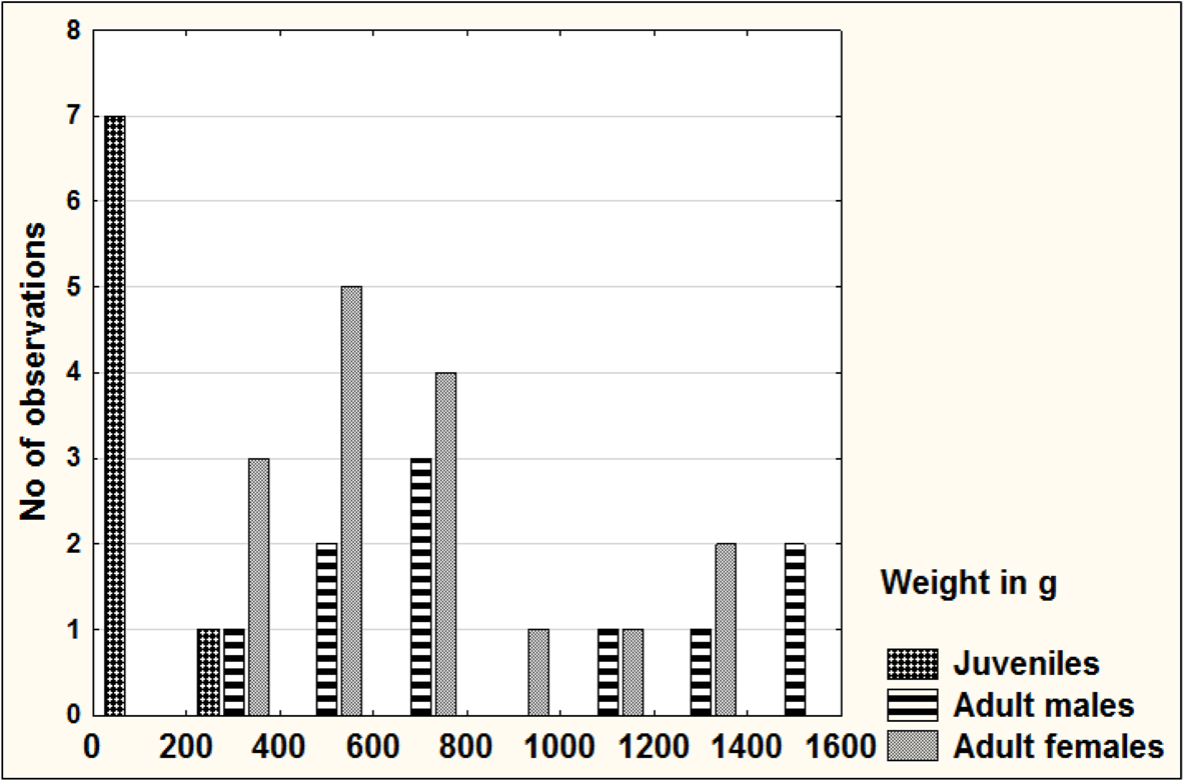
Distribution of weight among 34 blunt-nosed vipers from Cyprus

Morphometric data (Table 1) were collected on 34 vipers (10 adult males, 16 adult females and eight unsexed juveniles). Of these, 29 were alive and five (three males and two females) dead. No individuals were recaptured. Blunt-nosed viper rounded total lengths and weights (*n* = 34) ranged from 23.5 cm (10 g) for juveniles to 133.0 cm (1456 g) for a killed adult male (SNM-BS N-56085). The three largest males exceeded 130 cm ToL whereas the largest female (SNM-BS N-56086) had a rounded ToL of 124.0 cm. Rounded SVL ranged from 20.5 cm to 119.5 cm (Fig. 4). While the two heaviest males exceeded 1400 g body weight, the heaviest female, a gravid individual, weighed 1228 g (Fig. 5). The lowest (66) and highest (146) Fulton’s body condition index (K) was observed in adult females, whereas the range of K was much narrower among adult males (80–114) and juveniles (80–125). The mean ratio of TaL to ToL was 11.85 ± 1.18% for adult males (*n* = 10) and 10.80 ± 1.91% for adult females (*n* = 16). The coefficient of variation was highest for the variable weight, ranging from 46% in adult females to 113% in juveniles.

**Table 1:** Morphometric characteristics of juvenile and adult blunt-nosed vipers from Cyprus (SVL, TaL and ToL rounded to the nearest 0.5 cm)

Sex/Re-productive condition	Measurement	n	Range	Mean ± SD	Coefficient of Variation (%)
All	SVL (cm)	34	20.5–119.5	76.54 ± 28.30	36.97
	TaL (cm)	34	3.0–15.0	9.76 ± 3.51	35.93
	ToL (cm)	34	23.5–133.0	86.32 ± 31.49	36.47
	HL (mm)	32	16–54	40.59 ± 10.89	26.82
	HW (mm)	32	10–44	30.47 ± 9.21	30.24
	Weight (g)	34	10–1456	584.65 ± 432.05	73.90
	TaL / SVL	34	0.07–0.16	0.13 ± 0.02	15.65
	TaL / ToL	34	0.06–0.14	0.12 ± 0.02	14.28
	HL / SVL	32	0.04–0.10	0.06 ± 0.01	22.76
	HL / HW	32	1.18–1.70	1.36 ± 0.12	8.51
	BCI (K)	34	66–146	101.06 ± 18.41	18.21
Adult males	SVL (cm)	10	71.0–119.5	96.75 ± 18.93	19.57
	TaL (cm)	10	10.0–14.5	12.80 ± 1.40	10.93
	ToL (cm)	10	81.0–133.0	109.60 ± 20.24	18.47
	HL (mm)	10	40–54	48.60 ± 5.52	11.36
	HW (mm)	10	31–44	36.70 ± 4.67	12.72
	Weight (g)	10	346–1456	894.20 ± 420.79	47.06
	TaL / SVL	10	0.11–0.16	0.13 ± 0.02	11.17
	TaL / ToL	10	0.10–0.14	0.12 ± 0.01	9.92
	HL / SVL	10	0.05–0.06	0.05 ± 0.00	9.25
	HL / HW	10	1.23–1.42	1.33 ± 0.06	4.48
	BCI (K)	10	80–114	93.40 ± 9.99	10.70
Adult females	SVL (cm)	16	65.5–109.0	84.66 ± 12.47	14.73
	TaL (cm)	16	5.5–15.0	10.22 ± 2.30	22.53
	ToL (cm)	16	74.5–124.0	94.88 ± 13.74	14.48
	HL (mm)	14	36–48	43.71 ± 3.67	8.39
	HW (mm)	14	28–40	33.64 ± 3.69	10.97
	Weight (g)	16	279–1228	649.88 ± 300.90	46.30
	TaL / SVL	16	0.07–0.15	0.12 ± 0.02	19.20
	TaL / ToL	16	0.06–0.13	0.11 ± 0.02	17.65
	HL / SVL	14	0.04–0.06	0.05 ± 0.00	7.04
	HL / HW	14	1.18–1.47	1.30 ± 0.08	6.01
	BCI (K)	16	66–146	103.00 ± 22.43	21.77
Juveniles	SVL (cm)	8	20.5–59.5	35.06 ± 16.74	47.75
	TaL (cm)	8	3.0–8.5	5.06 ± 2.29	45.24
	ToL (cm)	8	23.5–68.0	40.13 ± 19.03	47.42
	HL (mm)	8	16–34	25.13 ± 8.72	34.73
	HW (mm)	8	10–24	17.13 ± 6.60	38.54
	Weight (g)	8	10–218	67.25 ± 76.04	113.06
	TaL / SVL	8	0.13–0.16	0.15 ± 0.01	4.58
	TaL / ToL	8	0.12–0.14	0.13 ± 0.01	3.99
	HL / SVL	8	0.06–0.10	0.08 ± 0.01	18.69
	HL / HW	8	1.33–1.70	1.49 ± 0.12	8.33
	BCI (K)	8	80–125	106.75 ± 16.18	15.15
Adults	SVL (cm)	26	65.5–119.5	89.31 ± 16.07	18.00
	TaL (cm)	26	5.5–15.0	11.21 ± 2.35	20.96
	ToL (cm)	26	74.5–133.0	100.54 ± 17.73	17.63
	HL (mm)	24	36–54	45.75 ± 5.06	11.06
	HW (mm)	24	28–44	34.92 ± 4.31	12.35
	Weight (g)	26	279–1456	743.85 ± 364.37	48.98
	TaL / SVL	26	0.07–0.16	0.13 ± 0.02	16.77
	TaL / ToL	26	0.06–0.14	0.11 ± 0.02	15.33
	HL / SVL	24	0.04–0.06	0.05 ± 0.00	7.90
	HL / HW	24	1.18–1.47	1.31 ± 0.07	5.37
	BCI (K)	26	66–146	99.31 ± 18.98	19.12

**Fig. 6:**
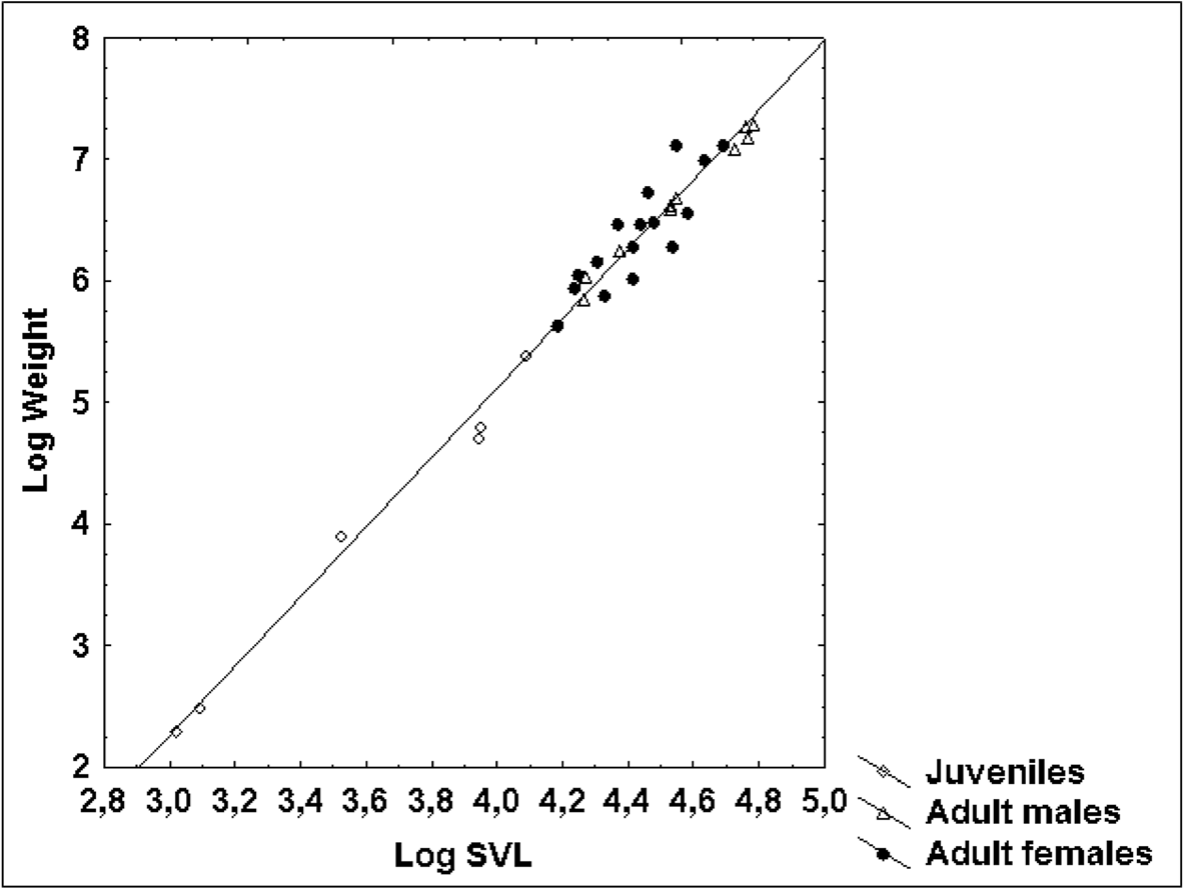
Relationship between log-transformed SVL and log-transformed weight in 34 blunt-nosed vipers from Cyprus: Y = − 6.32 + 2.86*X. R^2^ = 0.99

**Fig. 7:**
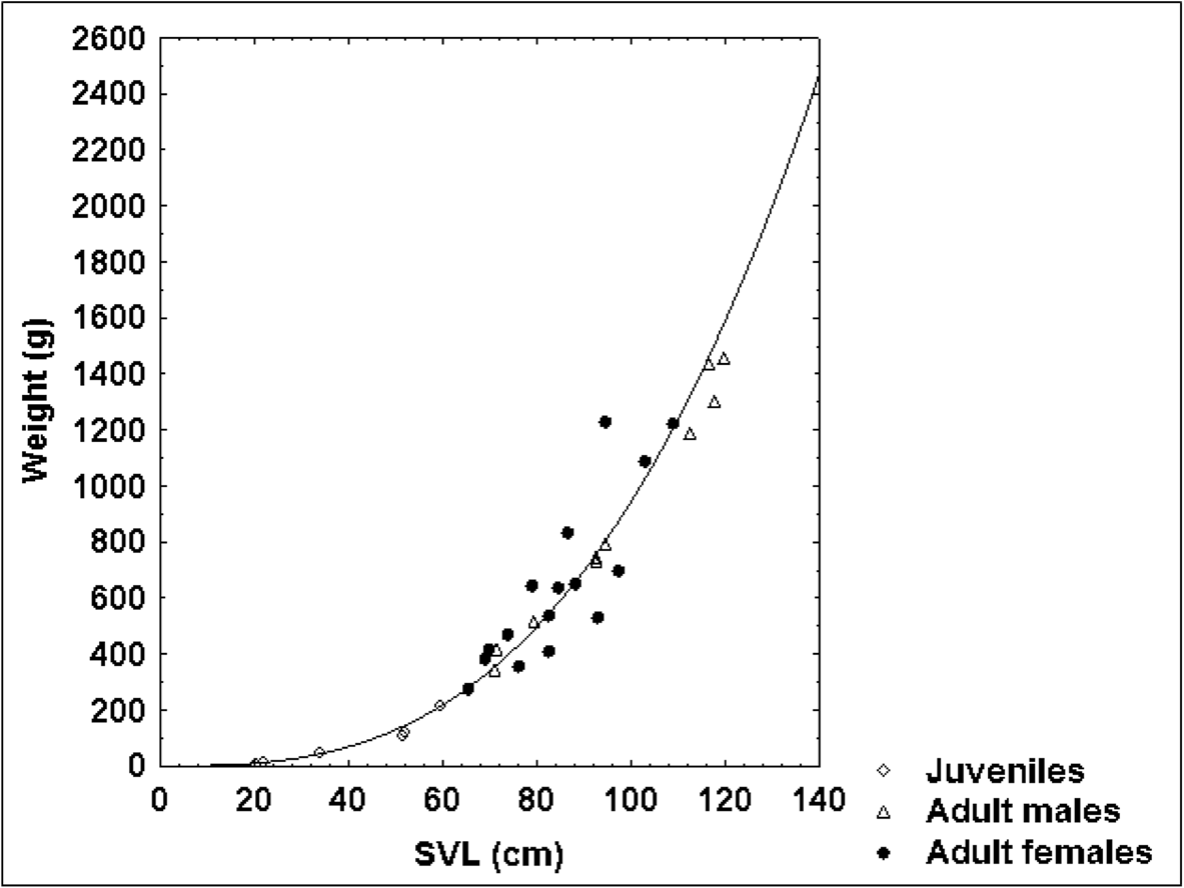
Allometric relationship between SVL and weight in 34 blunt-nosed vipers from Cyprus: Y = exp.(− 6.32)*X^286^

Figure 6 represents the relationship between natural logarithms of weight and natural logarithms of SVL for all examined vipers by a fitted regression line. The coefficient of determination R^2^ amounted to 0.99. In comparison, Fig. 7 demonstrates a relationship between original data with a fitted exponential function whose coefficients were estimated based on the linear regression approach. In adult females, the relationship between weight and SVL was much weaker (R^2^ = 0.73) than in juveniles and adult males (R^2^ = 0.97 and R^2^ = 0.99 respectively) (Table 2). Adult females also showed the weakest relationship between SVL and TaL, SVL and HL, SVL and HW, and between HL and HW (Table 3). The correlation between all characteristics was significant at the 0.05 level.

**Table 2:** Sample sizes, parameter and coefficient of determination (R^2^) of the regression: *logW* = *loga* + *b* ∙ *logSVL* of blunt-nosed vipers from Cyprus

	N	R^2^	a	b
SVL vs. weight (all)	34	0.80	−6.318	2.860
SVL vs. weight (juveniles)	8	0.97	−6.003	2.754
SVL vs. weight (adult males)	10	0.99	−4.961	2.557
SVL vs. weight (adult females)	16	0.73	−5.231	2.623

**Table 3:** Correlation coefficients (R) between morphometric characteristics of blunt-nosed vipers from Cyprus (significant *P* values bold)

	N	R	*P*_Correlation_
SVL vs. TaL (all)	33	0.93	**0.000**
SVL vs. TaL (juveniles)	8	0.997	**0.000**
SVL vs. TaL (adult males)	10	0.89	**0.001**
SVL vs. TaL (adult females)	15	0.58	**0.024**
SVL vs. HL (all)	32	0.97	**0.000**
SVL vs. HL (juveniles)	8	0.896	**0.003**
SVL vs. HL (adult males)	10	0.97	**0.000**
SVL vs. HL (adult females)	14	0.85	**0.000**
SVL vs. HW (all)	32	0.96	**0.000**
SVL vs. HW (juveniles)	8	0.93	**0.001**
SVL vs. HW (adult males)	10	0.93	**0.000**
SVL vs. HW (adult females)	14	0.72	**0.004**
HL vs. HW (all)	32	0.98	**0.000**
HL vs. HW (juveniles)	8	0.98	**0.000**
HL vs. HW (adult males)	10	0.93	**0.000**
HL vs. HW (adult females)	14	0.83	**0.000**

Morphometric characteristics SVL vs. TaL, SVL vs. HL, SVL vs. HW, and HL vs. HW of juveniles, adult males and adult females. For TaL, only undamaged tails were considered. All correlation coefficients were significant at the 0.05 level.

According to the *t* test, SVL, HW and weight were not significantly different between adult males and females at the 0.05 level, although *P* was relatively low: *P* values were calculated as 0.060, 0.087 and 0.097 for average SVL, HW and weight, respectively. Adult males were significantly longer in average TaL (*P* = 0.005), average ToL (*P* = 0.047) and average HL (*P* = 0.016). No significant sex-specific differences were found for HL / SVL, HL / HW and BCI (Table 4). The Mann-Whitney U test (Table 5) did not show a significant sex-specific difference for the ratios of TaL / SVL (*P* = 0.285) and TaL/ToL (*P* = 0.285). These results indicate that adult females did not have longer tails than adult males relative to their body length.

**Table 4:** Results of the *t* test for comparison of normally distributed morphometric characteristics of blunt-nosed vipers from Cyprus (significant *P* values bold)

Measurement	n adult males	n adult females	*P*
SVL (cm)	10	16	0.060
TaL (cm)	10	15	**0.005**
ToL (cm)	10	15	**0.047**
HL (mm)	10	14	**0.016**
HW (mm)	10	14	0.087
Weight (g)	10	16	0.097
HL / SVL	10	14	0.518
HL / HW	10	14	0.449
BCI (K)	10	16	0.216

**Table 5:** Results of the Mann-Whitney U test for comparison of not normally distributed morphometric characteristics of blunt-nosed vipers from Cyprus

Measurement	n adult males	n adult females	exact *P*
TaL / SVL	10	15	0.285
TaL / ToL	10	15	0.285

Adult females observed in the late summer of 2015 (*n* = 5) had a significantly lower mean BCI than those found in the spring of 2014 (*n* = 11) (*P* = 0.0007). Among adult males, the mean BCI was significantly lower in the four longest individuals (SVL ≥ 100 cm) than in the six smaller ones (SVL < 100 cm) (*P* = 0.013).

For measurements involving TaL, only individuals with undamaged tails were considered.

### Distance to water

Of 38 total field observations, 36 blunt-nosed vipers were recorded with corresponding distances to the nearest body of artificial and (or) natural freshwater in 2014 (*n* = 19) and in 2015 (*n* = 17) (Table 6). For a single observation in 2015, only the nearest distance to artificial water was recorded, decreasing the number of viper observations with both the nearest distance to artificial and to natural water bodies available by the value of 1 (2015: *n* = 16). Out of the 36 observed vipers, 24 individuals could be captured for data collection whereas 11 vipers either escaped or were killed by local people. In one case, a viper slough was treated as a single individual. At the site where a large male was observed, another large viper was recorded 21 days later, as were remains of a slough belonging to a similarly sized individual. To exclude the possibility of double-counting, all three observations were treated as one individual. Assumption of normality was rejected for all distances of *M. l. lebetina* to the nearest water bodies. The Mann-Whitney U test showed no significant difference between the distances of *M. l. lebetina* to the nearest water bodies in the spring of 2014 and late summer of 2015 (*P* = 0.537). Likewise, the distances of *M. l. lebetina* to natural water bodies in the spring of 2014 did not vary significantly from those in the late summer of 2015 (*P* = 0.497). There was also no significant difference between the distances of *M. l. lebetina* to artificial water bodies in the spring of 2014 and in the late summer of 2015 (*P* = 0.635). Equally, the Wilcoxon matched pairs test could not prove a significant difference between the distances of *M. l. lebetina* to natural and artificial water bodies in the spring of 2014 (*P* = 0.295) and in the late summer of 2015 (*P* = 0.717).

**Table 6:** Recorded distances of blunt-nosed vipers to the nearest water body (Paphos district, Cyprus)

Distances of blunt-nosed vipers	n	Range (meters)	Mean ± SD
To nearest water body (Spring 2014)	19	3–511	103.53 ± 143.82
To nearest natural water body (Spring 2014)	19	3–2111	485.95 ± 596.28
To nearest artificial water body (Spring 2014)	19	10–1092	211.74 ± 248.14
To nearest water body (Summer 2015)	17	0–1740	181.59 ± 416.38
To nearest natural water body (Summer 2015)	16	0–2853	864.50 ± 1038.65
To nearest artificial water body (Summer 2015)	17	0–2051	564.24 ± 675.22

## Discussion

### Habitat preferences

The summer season is a critical period for populations of *M. lebetina*. Female blunt-nosed vipers not only need to find suitable refuges for oviposition, incubation and clutch protection, but also must cope with postpartum weight loss. Newborn vipers face a diverse range of predators [8]. Due to their small body size, they are also more threatened by dehydration than adults [32]. Against this background, it is not surprising that Cypriot blunt-nosed vipers are often found close to freshwater [25, 33], particularly during the hot dry summer months [17]. Water preference has also been observed among *M. l. turanica* in Uzbekistan [34]. However, the present study could not prove a seasonal preference of *M. l. lebetina* for freshwater bodies. At the same time, the frequent observations of brown rats in all surveyed blunt-nosed viper habitats indicated sufficient prey availability in the study area. In fact, agricultural areas and other man-made landscapes with plenty of crops and organic waste offer conditions favorable to rat populations in Cyprus [25, 35, 36], which are large [37] and may attract blunt-nosed vipers even if freshwater is not available [38]. Besides ingesting the water contained in their prey animals, vipers can survive in xeric environments by drinking water from rain showers and mists. They can also harvest water from structural features of their environment such as rock surfaces after rain, or by dorsoventrally flattening their body to collect water droplets on their skin [39–41]. Snakes can further reduce their dehydration rate via resting in a coiled instead of stretched position [32]. Habitat structures allowing for thermoregulation and thermal protection have been proven critical for *M. l. obtusa*, and blunt-nosed vipers do not inhabit areas lacking these features [42]. In our current study, structural diversity in all surveyed habitats indicated an abundance of microhabitats providing shelter, humidity and thermoregulation to blunt-nosed vipers, also in areas void of freshwater bodies during summer. A 1993–1998 radio-telemetry study of the Cyclades blunt-nosed viper (*Macrovipera schweizeri*), now considered a subspecies of *M. lebetina* [43], found that this snake was not primarily attracted to water bodies but to structures that attracted their avian prey. These structures changed from water pools in spring to trees in autumn, when the pools had dried out. In both seasons, stream beds with riparian vegetation were important. *M. schweizeri* was absent from biotopes without large bushes as well as from densely vegetated habitats [44]. Our findings suggest that prey availability and protective microhabitat structures such as large bushes and rocks are critically important for preserving Cypriot blunt-nosed viper populations, whereas the annual drying out of water bodies in late summer does not seem to prevent *M. l. lebetina* from inhabiting areas. However, droughts have become more frequent on Cyprus [17], and their long-term impact on Cypriot snake species has not yet been studied (S. Zotos pers. comm. 12 March 2018).

### Shedding sites

Shedding increases dehydration in snakes [32]. As Cypriot blunt-nosed vipers slough four times a year, with two sloughings taking place during the summer months [17], the selection of suitable shedding sites is an important decision for the healthy development of *M. l. lebetina* throughout the year. Prior to molting, blunt-nosed vipers seek contact with water by bathing or lying in the rain to facilitate the sloughing of the old skin [8]. Water bodies may thus be particularly attractive for *M. l. lebetina* in pre-shed condition and may influence the selection of shedding locations, together with other factors (e.g. prey availability) that are communicated via chemical cues. The two blunt-nosed viper sloughs recorded in early April 2014 and late September 2015 were situated in habitats with year-round water availability, diversity of microstructures and abundance of brown rats. Based on observations of North American rattlesnakes (*Crotalus oreganus*), Loughran et al. (2015) pointed out that different social interactions (e.g., pairing and mating) occurred around shedding sites and that these places had a similar importance for the social structure of snakes as communal hibernacula [45]. This implies that water bodies are potentially important for facilitating interspecific contact in snakes, particularly in more arid environments such as Cyprus.

### Home range

Even though no data on *M. l. lebetina* movements are available, distances of 2–3 km to water bodies (as recorded for some individuals during this survey) may still fall within the home range of this species. Nilson et al. (1999) found that the home range of the smaller *M. schweizeri* was up to 20 ha, with one female covering a distance of 5 km within 12 months [44]. A radio-telemetry study in Cyprus would be highly useful for obtaining more precise information about *M. l. lebetina* habitat use.

### Artificial and natural water bodies

The annual drying-up of many natural water bodies in summer may imply that Cypriot blunt-nosed vipers prefer man-made, permanent water bodies during this season, but not necessarily during the rainier spring. Yet this study could neither confirm a preference for artificial over natural water bodies during late summer, nor could it provide evidence that vipers are found closer to artificial water bodies in late summer than in spring. These findings indicate that swimming pools are not more attractive for *M. l. lebetina* than natural pools during late summer, highlighting the importance of natural water bodies for blunt-nosed viper conservation. Since water bodies provide significant advantages for blunt-nosed vipers such as ambushing for prey [17], water uptake and thermoregulation during heat periods, swimming pool users still face an increased risk of viper encounters during summer. The viper bite incident in Latchi in September 2015 highlights the necessity of adequate protection measures. These include illuminating the entire pool area after sunset, wearing protective footwear, and cleaning debris from the surroundings, as blunt-nosed vipers (especially juveniles) may hide underneath larger, flat-surfaced objects such as wooden boards and flower pots (pers. obs.). Vipers can be kept away from properties by solid fences whereas nets will indiscriminately kill various species of reptiles and other wildlife by entanglement [46]. The idea of developing effective snake repellents is promising, but commercially available chemicals such as naphthalene and sulfur have not proven effective in field trials [47].

### Anthropogenic impact and conservation

The ongoing anthropogenic destruction of wild snake habitats for real-estate and industrial development in Cyprus [17] may threaten the survival of wild *M. l. lebetina* populations and increase the likelihood of human-viper confrontations in the future. Habitat loss due to land transformation for tourism is a general threat to snake species on Eastern Mediterranean islands [48]. Wildfires, which regularly occur in Cyprus, may render habitats unsuitable for snakes and their prey organisms (e.g. birds) for up to 10 years. Together with surface mining, wildfires are a major threat to blunt-nosed viper habitats on the island of Milos [44]. Road traffic is a threat to *M. lebetina* throughout the species’ range [8].

Although *M. l. lebetina* is listed in Appendix II of the Berne convention (which contains “strictly protected fauna species”), it is the only snake species not protected by law in Cyprus [17], but at the same time the most pursued one. As all surveyed water bodies were checked at least monthly by mosquito control workers, the local viper populations were exposed to continuous anthropogenic pressure (Fig. 3d). This ongoing human encroachment of riparian habitats coupled with the consequent killing of blunt-nosed vipers may greatly reduce their populations along streams and can be regarded as another major threat to the species in Cyprus (H.-J. Wiedl pers. comm. 29 March 2014). Pursuit by hunters must be considered as a significant threat, too. In Cyprus, feral cats commonly prey on reptiles, including *Laudakia stellio* and *Dolichophis jugularis* [17]. Feral cat predation on young vipers also poses a threat to the reproduction of *M. schweizeri* on Milos [44]. Consequently, their impact on *M. l. lebetina* should be investigated.

Workshops for mosquito control workers and other (occupational) outdoor groups such as hunters, shepherds, farmers, forestry employees and game wardens could help to raise awareness of non-lethal methods of preventing human-viper conflict such as snake deterrence or translocation. Workshops should also be offered to rural communities and schools. Public education could be further aided by the establishment of a national snakebite database for Cyprus, which so far is not available (E. Erotokritou pers. comm. 12 October 2017). Valuable snake habitats such as wild riparian areas and rocky slopes with confirmed populations of *M. l. lebetina* should be placed under protection, with prohibition of land transformation (e.g., for real-estate development), and strict regulations concerning further interventions by man (e.g., grazing). This could be achieved by designating new areas for the Natura 2000 network of the European Union. In all conservation areas, hunting should be prohibited. Wetlands are also important habitats of other endangered Cypriot reptiles such as *Hierophis cypriensis* (Cyprus whip snake), *Natrix natrix cypriaca* (grass snake) and *Mauremys rivulata* (Balkan terrapin) [17].

### Allometric relationship between snout-vent length and weight

The allometric relationship between SVL and weight in *M. l. lebetina* (*n* = 34) can be modelled as an exponential curve (Fig. 7) fitted to the scatterplot of SVL vs. weight. According to the equation, a very large Cypriot blunt-nosed viper of 135 cm SVL would weigh 2227 g, which is not far from the reported maximum body weight of mainland blunt-nosed vipers according to Ščerbak & Böhme (2005): 2700 g for males and 2000 g for females [8]. However, Sochurek (1979) reports that he obtained a large male blunt-nosed viper of more than 3 kg body weight from the USSR around the year 1950 [49]. Out of 23 blunt-nosed vipers collected in Iran (Khorasan province), the heaviest male (SVL 125.5 cm, TaL 17.0 cm) and female (SVL 113.0 cm, TaL 14.0 cm) each weighed 1180 g ([50], A. Nasoori pers. comm. 15 January 2018). While Cypriot blunt-nosed vipers can reach a ToL of 150 cm [17], mainland subspecies (e.g., *M. l. obtusa*) may grow up to 218 cm [51]; however, one individual of 230 cm was recorded [18]. Bannikov et al. (1977) state a maximum ToL of 160 cm [52], whereas Sochurek (1979) mentions specimens of up to 190 cm ToL [49].

### Variability of body condition

**Fig. 8:**
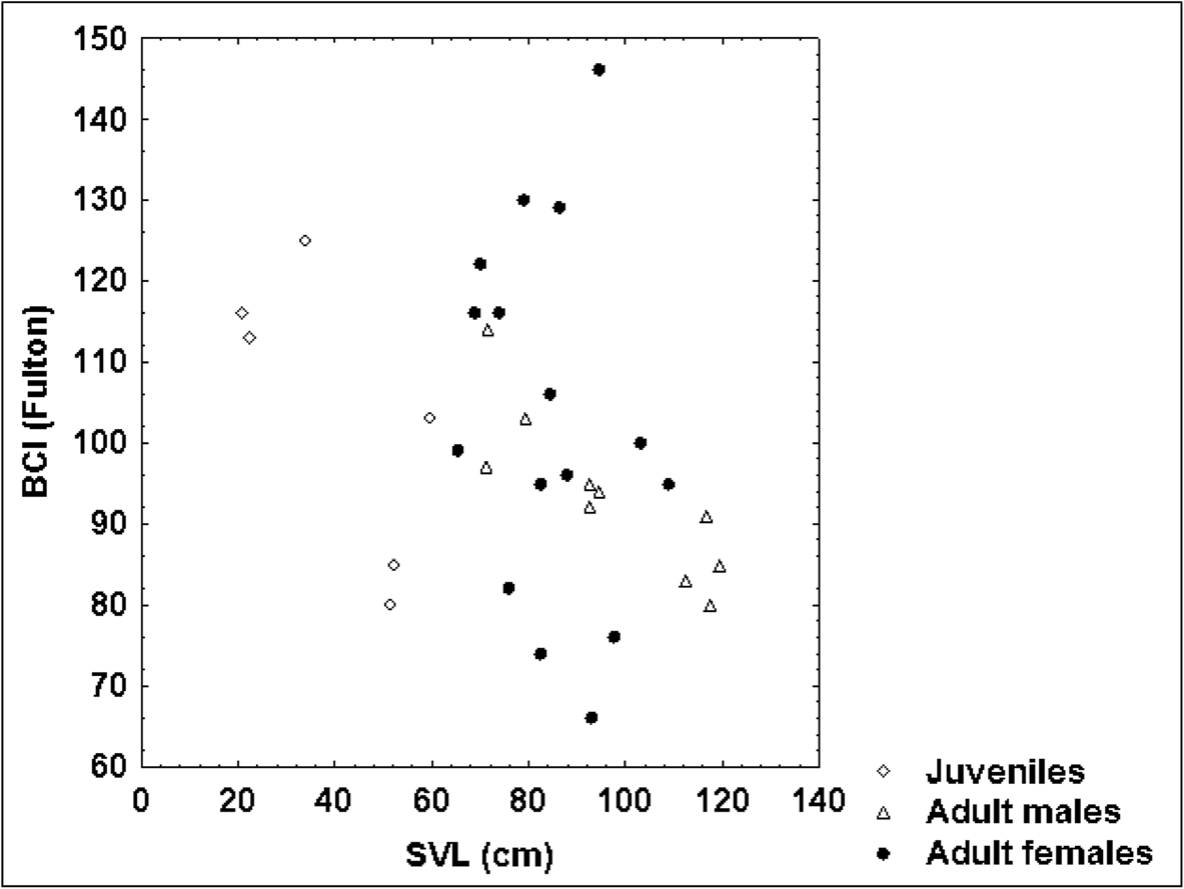
Relationship between SVL and body condition index (BCI) in 34 blunt-nosed vipers from Cyprus: *R* = 0.40

The highest coefficient of variation among all variables in this study was determined for weight (from 46% in adult females to 113% in juveniles). It corresponds to the identified positive allometric relationship between weight and the linear measures of the blunt-nosed viper’s body. The relatively low R^2^ determination coefficient of the regression ln weight vs. ln SVL among adult female blunt-nosed vipers (0.73) shows that the body weight of females was not only influenced by their SVL but also by other factors. In line with our hypothesis, adult *M. l. lebetina* females caught in September were significantly thinner than those captured in spring (*P* = 0.001). This fluctuation of body mass might be explained by postpartum weight loss, provided that the females examined in late summer had reproduced in that year. After oviposition, blunt-nosed vipers (*M. l. obtusa*) may lose up to 52% of their weight (mean: 35%) [53]. Snake body condition is also influenced by the size and time of the last meal, and by diseases or parasites. Adult male blunt-nosed vipers are more likely to thrive during late summer than during spring, as they neither have to search for females nor engage in combat with male rivals anymore [17]. However, the low number of adult males recorded during this study in late summer (three compared to seven males in spring) prevented a seasonal comparison within males. Although our sample of adult male vipers was small (*n* = 10), the significantly lower body condition index of the four largest adult males compared to the six smaller ones was striking (*P* = 0.013), since it suggests that male blunt-nosed vipers may become thinner with age. This hypothesis would be consistent with the trend of the scatterplot of SVL vs. BCI (Fig. 8) showing that the highest BCI values were observed among small and medium-sized vipers (SVL < 100 cm). Large blunt-nosed vipers may lose weight due to the accumulation of endoparasites in snakes with increasing age, and the consequent loss of appetite and weight [54–56]. Zinyakova (1967) detected 25 species of endoparasites in *M. l. turanica* from the Soviet Union [57], while Murvanidze et al. (2008) listed seven species of helminths inhabiting Georgian *M. l. obtusa* [58]. Of 20 Iranian blunt-nosed vipers (*M. l. obtusa*) examined by Nasiri et al. (2014), 16 were infested with parasites [59]. As our sample included only two females exceeding 100 cm SVL, a comparison between larger and smaller adult females was impossible (14:2 ratio). Our findings indicate that adult female blunt-nosed vipers are in a more vulnerable physical condition in late summer than in spring, and that old age possibly has an adverse effect on the body condition index of *M. l. lebetina*. However, a much larger dataset is needed to provide solid statistical evidence.

### Sex-specific differences

**Fig. 9:**
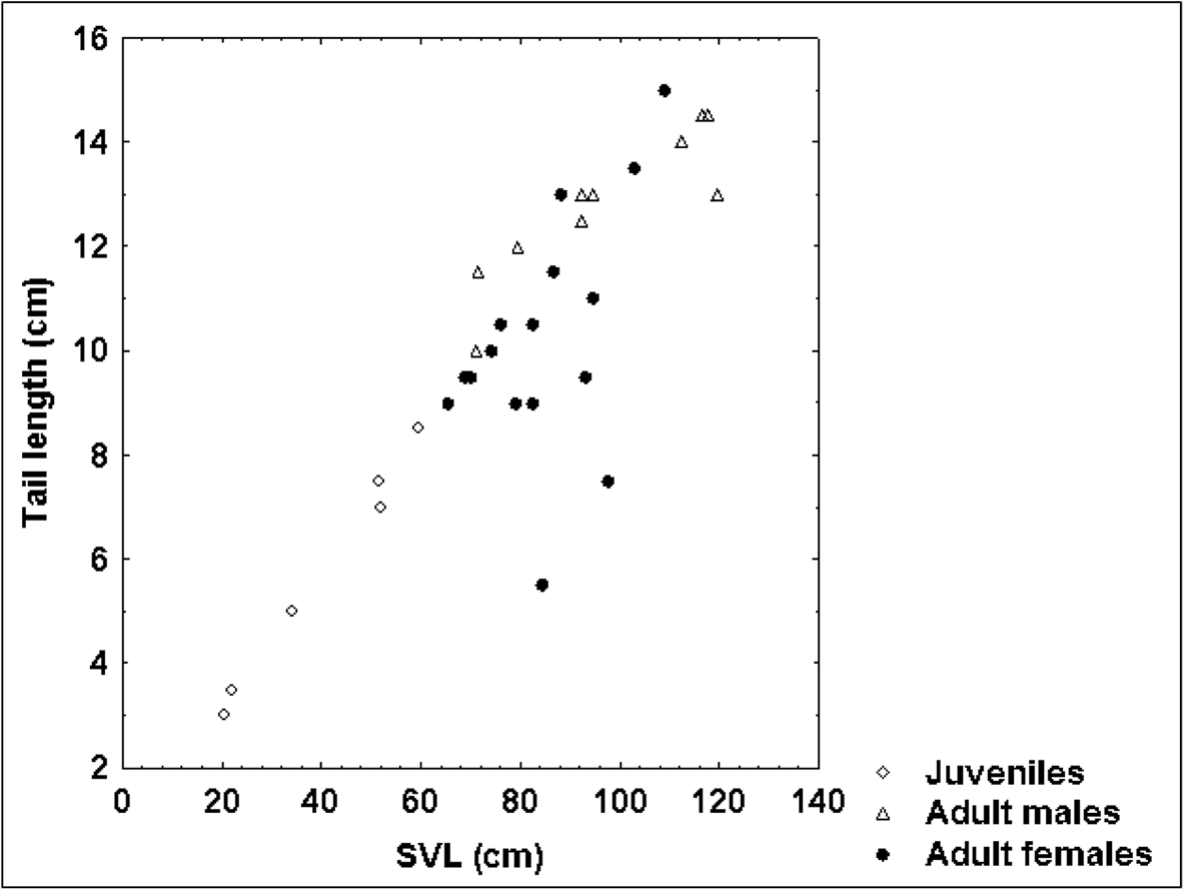
Relationship between SVL and TaL in 34 blunt-nosed vipers from Cyprus: *R* = 0.89

**Fig. 10:**
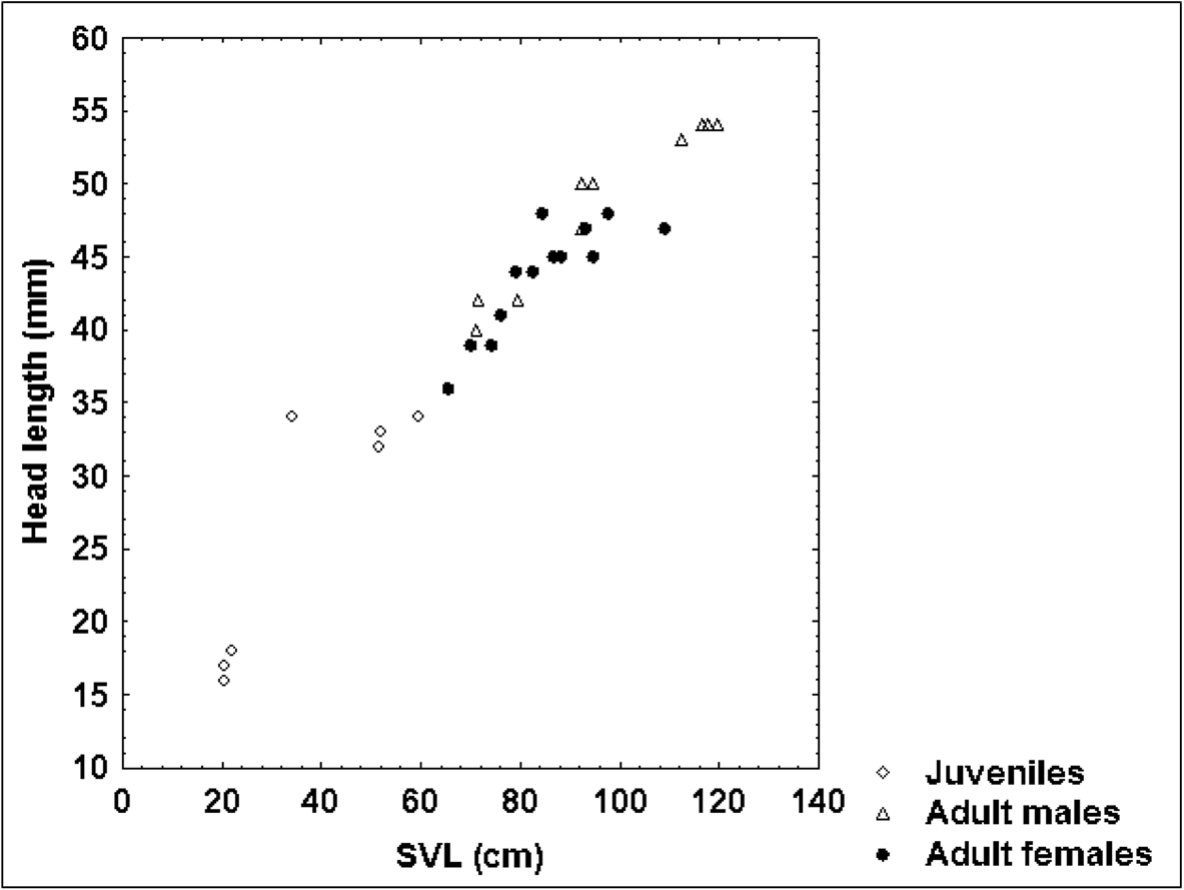
Relationship between SVL and HL in 32 blunt-nosed vipers from Cyprus: *R* = 0.97

**Fig. 11:**
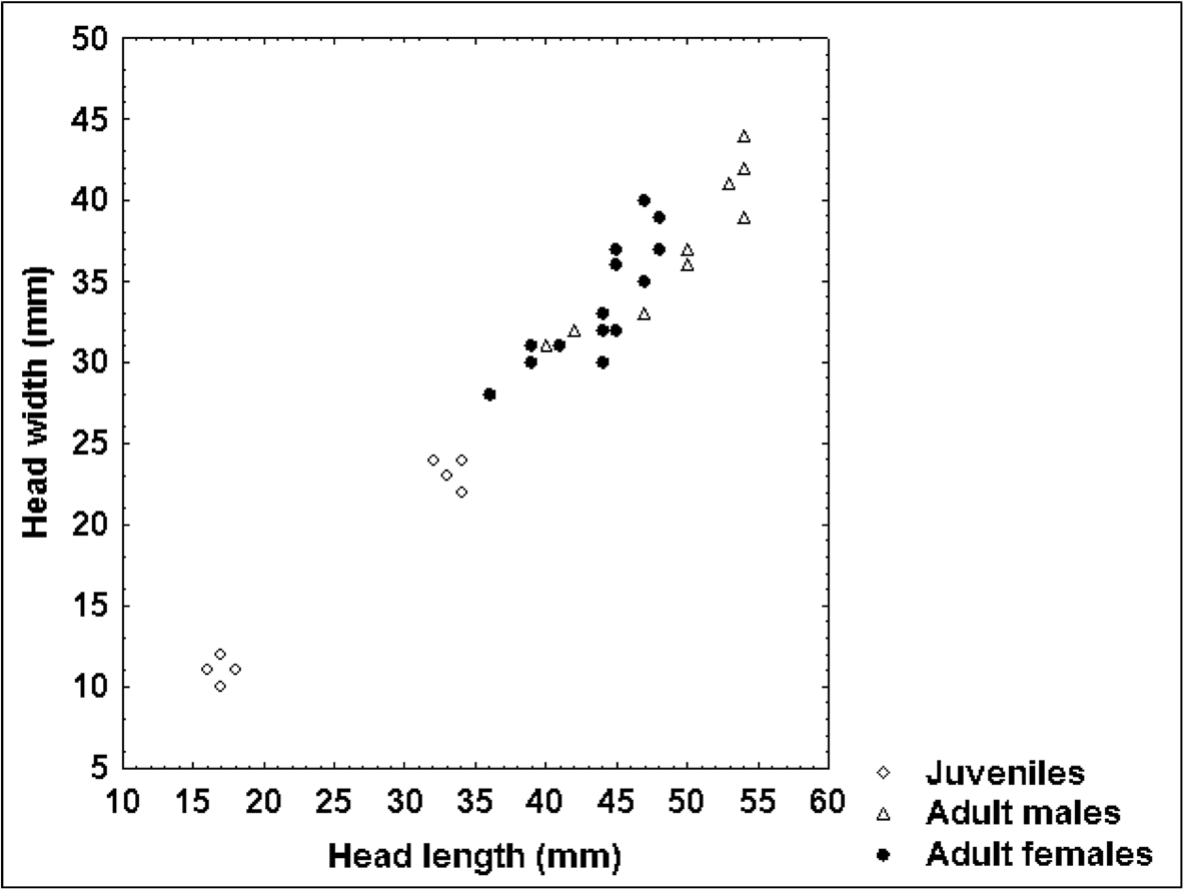
Relationship between HL and HW in 32 blunt-nosed vipers from Cyprus: *R* = 0.98

Although we could not prove that adult male blunt-nosed vipers exceeded adult females in body size and weight (*P*-values of the respective t test amounted to 0.06 for SVL and 0.097 for weight), our results suggested that they were longer and heavier. This apparent dimorphism was further supported by the significantly longer TaL (*P* = 0.005), ToL (*P* = 0.047) and HL (*P* = 0.016) of adult males. This trend is also shown by the scatterplots of SVL vs. TaL (Fig. 9), SVL vs. HL (Fig. 10) and HL vs. HW (Fig. 11). As the ratio of TaL to SVL and of TaL to ToL in adult individuals was not significantly different between sexes, we could not confirm our hypothesis that males had shorter tails than females (see [17]). This indicates that sex-specific morphological differences in *M. l. lebetina* are still a controversial issue. However, our findings of the ratio TaL / ToL were similar to those of other authors [50], who recorded a mean ratio of 11.89 ± 1.56% among 15 adult males (our study: 11.85 ± 1.18%) and a mean ratio of 11.10 ± 0.59% in eight adult females of Iranian *M. l. obtusa* (our study: 10.80 ± 1.91%). Larger sample sizes are required to obtain more powerful test results on sex-specific differences.

### Size comparison with other large Holarctic vipers

The enormous differences in body size between blunt-nosed vipers from the Cyclades and Cyprus [8, 17, 44, 60] demonstrate the morphological variability within *M. lebetina*. It is the only European viper reaching 150 cm ToL and 2000 g body mass (or more), which is comparable to the body dimensions of large North American rattlesnake species (*Crotalus* spp.) (Table 7). Some of the latter (e.g., *C. atrox*) are found in similar biomes (dry steppe, semiarid desert) as *M. lebetina*. In the Mediterranean basin, Moorish vipers (*Daboia mauritanica*) and desert vipers (*D. deserti*) reach similar dimensions (180 cm and 160 cm maximum ToL, respectively), whereas Palestine (*D. palaestinae*) and Ottoman vipers (*Montivipera xanthina*) commonly do not grow much larger than 1 m [61]. However, exceptionally long individuals may reach 136 cm ToL (1227 g body mass) in *D. palaestinae* (S. Meiri pers. comm. 1 May 2017) and 143.5 cm ToL (1284 g body mass) in *M. xanthina nilsoni* ([62]; A. Cattaneo pers. comm. 2 August 2017). Maximum recorded body weights of *D. palaestinae* were 1640 g (131 cm ToL) and 1800 g (128 cm ToL), respectively, the latter being a gravid female (S. Meiri, unpublished data). As large viper species are primarily found in the Americas, Africa, the Middle East and South Asia, the enormous body dimensions of *M. l. lebetina* highlight its morphological and evolutionary uniqueness within the European viper fauna.

**Table 7:** Snout-vent length, weight and body condition index (BCI) of *M. l. lebetina* compared to large North American rattlesnake species (*Crotalus* spp., USA)

General information [31, 61]					
Scientific name	*M. lebetina*	*C. adamanteus*	*C. atrox*	*C. horridus*	*C. oreganus*
Common name	Blunt-nosed viper	Eastern diamondback rattlesnake	Western diamondback rattlesnake	Timber rattlesnake	Western rattlesnake
Distribution	Eurasia to Central and South Asia	Southeastern USA	Southwestern North America	Eastern to Central USA	Western North America
Maximum ToL (cm)	150 (Cyprus); 218–230 (mainland)	244	213	189	163
Specimen data					
Origin	Wild, Cyprus	Captive, North Carolina	Wild, Oklahoma	Wild, Virginia	Wild, California
SVL (cm)	116.5	168.8	152.0	140.7	109.0
TaL (cm)	14.5	16.0	–	11.3	9.0
Weight (g)	1441	4850 (“4.85 kg”)	2776	3509	1150
BCI (K)	91	101	79	126	89
Reference	This study	[63]	[64]	S. Goetz pers. comm. 29 January 2018	B. Putman pers. comm. 19 January 2018

## Conclusions

We conclude that adult males of *M. l. lebetina* exceed adult females in head length, tail length and total length. Adult female blunt-nosed vipers are probably in a more vulnerable physical condition in late summer than in spring. Old age possibly has an adverse effect on the body condition index of *M. l. lebetina*. The annual drying out of freshwater bodies in late summer most likely is not a decisive factor for the occurrence of the species on Cyprus. It must be assumed that prey availability and protective microhabitat structures such as large bushes and rocks are of crucial importance for preserving blunt-nosed viper populations in Cyprus. A radio-telemetry study would greatly improve the understanding of the spatial ecology of *M. l. lebetina*. Workshops should be conducted for rural communities, schools and outdoor groups to raise awareness of non-lethal ways of removing blunt-nosed vipers from areas inhabited or frequented by people. The establishment of a national snakebite database for Cyprus would contribute to improving public education. Wild snake habitats with confirmed populations of *M. l. lebetina* should be protected from anthropogenic modifications such as real-estate development, grazing and hunting.

## Abbreviations

°C: Degrees Celsius
°E: Eastern latitude
°N: Northern latitude
AM: Time before noon
BCI (K): Fulton’s body condition index
cm: Centimeters
CV: Coefficient of variation
Fig.: Figure
g: Grams
HL / HW: Ratio of head length to head width
HL / SVL: Ratio of head length to snout-vent length
HL: Head length
HW: Head width
kg: Kilograms
m: Meters
mm: 1) millimeters of length, 2) millimeters of precipitation
n: Sample size
*P*: Probability value
PM: Time after noon
R: Correlation coefficient
R^2^: Coefficient of determination
SD: Standard deviation
SVL: Snout-vent length
TaL / SVL: Ratio of tail length to snout-vent length
TaL / ToL: Ratio of tail length to total length
TaL: Tail length
ToL: Total length
W: Weight in grams

## References

[1] Corkill NL (1932). Snakes of Iraq. J Bombay Nat Hist Soc.

[2] Stümpel N, Joger U, Neubert E, Amr Z, Taiti S, Gümüs B (2009). Recent advances in phylogeny and taxonomy of near and middle eastern vipers - an update. Animal biodiversity in the Middle East: proceedings of the first middle eastern biodiversity congress; 20–23 October 2008.

[3] Cesaretli Y, Ozkan O (2010). Snakebites in Turkey: epidemiological and clinical aspects between the years 1995 and 2004. J Venom Anim Toxins incl Trop Dis..

[4] Sagheb MM, Sharifian M, Moini M, Salehi O (2011). Acute renal failure and acute necrotizing pancreatitis after *Echis carinatus sochureki* bite, report of a rare complication from Southern Iran. Prague Med Rep.

[5] Dehghani R, Mehrpour O, Shahi MP, Jazayeri M, Karrari P, Keyler D (2014). Epidemiology of venomous and semi-venomous snakebites (Ophidia: Viperidae, Colubridae) in the Kashan city of the Isfahan province in Central Iran. J Res Med Sci.

[6] Swaroop S, Grab B (1954). Snakebite mortality in the world. Bull World Health Organ.

[7] Hopkins GO (1974). Snake bites in Cyprus. J R Army Med Corps.

[8] Ščerbak N, Böhme W, Joger U, Stümpel N (2005). *Macrovipera lebetina* (Linnaeus, 1758) - Levante-otter. Handbuch der Reptilien und Amphibien Europas. Band 3/IIB, Schlangen III.

[9] Sharma LR, Lal V, Simpson ID (2008). Snakes of medical significance in India: the first reported case of envenoming by the Levantine viper (*Macrovipera lebetina*). Wilderness Environ Med.

[10] Phelps T (2010). Old world vipers. A natural history of the Azemiopinae and Viperinae.

[11] Göçmen B, Arikan H, Ozbel Y, Mermer A, Çiçek K (2006). Clinical, physiological and serological observations of a human following a venomous bite by *Macrovipera lebetina lebetina* (Reptilia: Serpentes). Turkiye Parazitol Derg.

[12] Bazaa A, Marrakchi N, El Ayeb M, Sanz L, Calvete JJ (2005). Snake venomics: comparative analysis of the venom proteomes of the Tunisian snakes *Cerastes cerastes*, *Cerastes vipera* and *Macrovipera lebetina*. Proteomics.

[13] Park MH, Jo M, Won D, Song HS, Han SB, Song MJ (2012). Snake venom toxin from *Vipera lebetina turanica* induces apoptosis of colon cancer cells via upregulation of ROS- and JNK-mediated death receptor expression. BMC Cancer.

[14] Nalbantsoy A, Karabay-Yavasoglu NU, Sayım F, Deliloglu-Gurhan I, Göçmen B, Arıkan H (2012). Determination of in-vivo toxicity and in-vitro cytotoxicity of venom from the Cypriot blunt-nosed viper *Macrovipera lebetina lebetina* and antivenom production. J Venom Anim Toxins incl Trop Dis.

[15] Ozen MO, İğci N, Yalçin HT, Göçmen B, Nalbantsoy A (2015). Screening of cytotoxic and antimicrobial activity potential of Anatolian *Macrovipera lebetina obtusa* (Ophidia: Viperidae) crude venom. Front Life Sci.

[16] Suzergoz F, İğci N, Çavuş C, Yildiz MZ, Coşkun MB, Göçmen B (2016). In vitro cytotoxic and proapoptotic activities of Anatolian *Macrovipera lebetina obtusa* (Dwigubski, 1832) crude venom on cultured K562 human chronic myelogenous leukemia cells. Int J Hematol Oncol.

[17] Baier F, Sparrow DJ, Wiedl HJ (2013). The amphibians and reptiles of Cyprus.

[18] Mermer A, Göçmen B, Çiçek K (2012). Extreme cases of colour pattern and size in Levantine viper, *Macrovipera lebetina* (L., 1758) from the west of Euphrates Basin (southern Anatolia, Turkey). Biharean Biol.

[19] Gruber U (1989). Die Schlangen Europas und rund ums Mittelmeer.

[20] Feldman A, Meiri S (2013). Length-mass allometry in snakes. Biol J Linn Soc.

[21] Băncilă RI, Hartel T, Plăiașu R, Smets J, Cogălniceanu D (2010). Comparing three body condition indices in amphibians: a case study of yellow-bellied toad *Bombina variegata*. Amphib Reptil..

[22] Sahlean TC, Strugariu A, Dincă PC, Chișamera G, Stanciu CR, Zamfirescu ȘR (2016). Morphological characteristics of the elusive blotched snake (*Elaphe sauromates*) at its northwestern range limit (Romania). Turkish J Biol.

[23] Tsairi H, Bouskila A (2004). Ambush site selection of a desert snake (*Echis coloratus*) at an oasis. Herpetologica.

[24] García-Cardenete L, Pleguezuelos JM, Brito JC, Jiménez-Cazalla F, Pérez-García MT, Santos X (2014). Water cisterns as death traps for amphibians and reptiles in arid environments. Environ Conserv.

[25] Kabisch K, Wiedl HJ (2009). Zur Levanteotter, *Macrovipera lebetina lebetina* (Linnaeus, 1758) auf Zypern. Sauria.

[26] Constantinides G (2002). Coastal area management Programme Cyprus: diagnostic - feasibility report.

[27] Delipetrou P, Makhzoumi J, Dimopoulos P, Georghiou K, Vogiatzakis I, Pungetti G, Mannion AM (2008). Cyprus. Mediterranean Island landscapes - natural and cultural approaches.

[28] Climatological data, Polis, 1991-2005. Cyprus Department of Meteorology, Nicosia. - [cited 24 May 2018]. Available from: http://www.moa.gov.cy/moa/MS/MS.nsf/All/FD3278466EACCF9FC22576C80036B9DF/$file/Climatological%20Data_1991_2005_Polis%20Chrysochous_U.K.pdf?Openelement.

[29] Shine R, Webb JK, Fitzgerald M, Sumner J (1998). The impact of bush-rock removal on an endangered snake species, *Hoplocephalus bugaroides* (Serpentes: Elapidae). Wildl Res.

[30] Benson PA (1999). Identifying individual adders, *Vipera berus*, within an isolated colony in East Yorkshire. Herpetol Bull.

[31] Klauber LM (1972). Rattlesnakes: their habits, life histories, and influence on mankind.

[32] Cohen AC (1975). Some factors affecting water economy in snakes. Comp Biochem Physiol.

[33] Osenegg K (1989). Die Amphibien und Reptilien der Insel Zypern [diploma thesis].

[34] Cherlin VA, Shepilov SA (2014). Thermal biology of the central Asian blunt-nosed viper (*Macrovipera lebetina turanica*) from Nuratau crest and the Chernov blunt-nosed viper (*Macrovipera lebetina černovi*) from Western Kysylkum. Biol Bull.

[35] Watson J. The rat problem in Cyprus, Col. Res. Publ. 1951;9:1–66.

[36] Davies WNL (1970). The carob tree and its importance in the agricultural economy of Cyprus. Econ Bot.

[37] Psaroulaki A, Antoniou M, Toumazos P, Mazeris A, Ioannou I, Chochlakis D (2010). Rats as indicators of the presence and dispersal of six zoonotic microbial agents in Cyprus, an island ecosystem: a seroepidemiological study. Trans R Soc Trop Med Hyg.

[38] Manteuffel D (1993). Bericht über Reptilienfunde in der Türkei. Salamandra.

[39] Saint-Girons H, Bauchot R (1994). Physiologie. Schlangen.

[40] Repp RA, Schuett GW (2008). Western diamond-backed rattlesnake, *Crotalus atrox* (Serpentes: Viperidae), gain water by harvesting and drinking rain, sleet, and snow. Southwest Nat.

[41] Glaudas X (2009). Rain-harvesting by the southwestern speckled rattlesnake (*Crotalus mitchellii pyrrhus*). Southwest Nat.

[42] Iskenderov TM, Javadov SA (2013). Some aspects of thermobiology of the south Caucasian Gyurza (*Macrovipera lebetina obtusa* Dwigubsky, 1832). J Entomol Zool Stud.

[43] Stümpel N (2012). Phylogenie und Phylogeographie eurasischer Viperinae unter besonderer Berücksichtigung der orientalischen Vipern der Gattungen *Montivipera* und *Macrovipera* [dissertation].

[44] Nilson G, Andrén C, Ioannidis Y, Dimaki M (1999). Ecology and conservation of the Milos viper, *Macrovipera schweizeri* (Werner, 1935). Amphib Reptil.

[45] Loughran CL, Beck DD, Weaver RE (2015). Use of communal shedding sites by the northern pacific rattlesnake (*Crotalus oreganus oreganus*) in Central Washington state. Northwest Nat.

[46] Ferronato BO, Roe JH, Georges A (2014). Reptile bycatch in a pest-exclusion fence established for wildlife reintroductions. J Nat Conserv.

[47] Ferraro DM, Masters RE, Huggins JG (1995). The efficacy of naphthalene and sulfur repellents to cause avoidance behavior in the plains garter snake. Twelfth Great Plains wildlife damage control workshop proceedings; Tulsa, Oklahoma.

[48] Nichol J (1989). Bites and stings. The world of venomous animals.

[49] Sochurek E (1979). Kritische Liste der Giftschlangen Europas mit Beschreibung einer neuen Unterart. Mitt Zool Ges Braunau.

[50] Nasoori A, Taghipour A, Shahbazzadeh D, Aminirissehei A, Moghaddam S (2014). Heart place and tail length evaluation in *Naja oxiana*, *Macrovipera lebetina*, and *Montivipera latifii*. Asian Pac J Trop Med.

[51] Herrmann HW, Joger U, Nilson G (1992). Phylogeny and systematics of viperine snakes. III: resurrection of the genus *Macrovipera* (Reuss, 1927) as suggested by biochemical evidence. Amphib Reptil..

[52] Bannikov AG, Darevsky IS, Išeenko VG, Rustamov AK, Ščerbak NN (1977). Opredelitel zemnovodnikh i presmikajuschikhsya fauni SSSR.

[53] Korneva LG (1972). The reproduction of *Vipera lebetina*. Zool Zh.

[54] Lenz S (1994). Beobachtungen zum Paarungsverhalten und zum Parasitenbefall der Puffotter (*Bitis arietans*) in Gambia/Westafrika. Salamandra..

[55] Junker K, Lane EP, Dlamini B, Kotze A, Boomker J (2009). *Post mortem* identification of *Kalicephalus colubri colubri* (Nematoda: Diaphanocephalidae) in a captive mole snake (*Pseudapis cana*) in South Africa. J S Afr Vet Assoc.

[56] Kavitha KT, Latha BR, Bino Sundar ST, Jayathangaraj MG, Senthil Kumar K, Sridhar R (2013). *Kalicephalus* sp. in a captive Russell’s viper: a case report. J Parasit Dis.

[57] Zinyakova MP (1967). Rasprostanenie, ecologia gurzi (Vipera lebetina turanica Černov) i soderzanie eje v serpentarii.

[58] Murvanidze L, Lomidze TS, Nikolaishvili K, Jankarashvili E, Bakhtadze GI, Beltadze N, Bukhnikashvili A, Eliava IJ, Japoshvili G, Kvavadze S, Melashvili NO, Murvanidze M, Tarkhnishvili ED (2008). The annotated list of reptile helminths of Georgia. Proceedings of the Institute of Zoology, volume XXIII; 2008; Tbilisi, Georgia.

[59] Nasiri V, Mobedi I, Dalimi A, Mirakabadi AZ, Ghaffarifar F, Teymurzadeh S (2014). A description of parasites from Iranian snakes. Exp Parasitol.

[60] Nilson G, Joger U, Stümpel N (2005). *Macrovipera schweizeri* (WERNER, 1935) - Kykladenviper. Handbuch der Reptilien und Amphibien Europas. Band 3/IIB, Schlangen III.

[61] Trutnau L (1998). Giftschlangen. Schlangen im terrarium - band 2.

[62] Cattaneo A (2014). Variabilita e sottospecie di *Montivipera xanthina* (Gray, 1849) nelle isole egee orientali (Reptilia Serpentes Viperidae). Nat sicil.

[63] Palmer WA, Braswell AL (1995). Reptiles of North Carolina.

[64] Fitch HS, Pisani GR (1993). Life history traits of the western diamondback rattlesnake (*Crotalus atrox*) studied from roundup samples in Oklahoma. Occ Pap Mus Nat Hist Univ Kansas.

